# The Influence of Monitoring Interval on Data Measurement: An Analysis of Step Counts of University Students

**DOI:** 10.3390/ijerph10020515

**Published:** 2013-01-28

**Authors:** Dagmar Sigmundová, Jana Vašíčková, Jiří Stelzer, Emil Řepka

**Affiliations:** 1 Center for Kinanthropology Research, Institute of Active Lifestyle, Faculty of Physical Culture, Palacky University in Olomouc, Tr. Miru 115, Olomouc 77111, Czech Republic; E-Mail: jana.vasickova@upol.cz; 2 Department of Kinesiology and Physical Education, Valdosta State University, 1500 N Patterson St., Valdosta, GA 31698, USA.; E-Mail: jstelzer@valdosta.edu; 3 Faculty of Education, University of South Bohemia, Ceske Budejovice, Jeronymova 10, Ceske Budejovice 37115, Czech Republic.; E-Mail: repka@pf.jcu.cz

**Keywords:** reliability, intra-class correlation, pedometer, steps, youth

## Abstract

The pedometer is a widely used research tool for measuring the level and extent of physical activity (PA) within population subgroups. The sample used in this study was drawn from a population of university students to examine the influence of the monitoring interval and alternate starting days on step-count activity patterns. The study was part of a national project during 2008–2010. Eligible subjects (641) were selected from a sample of 906 university students. The students wore pedometers continuously for 7 days excluding time for sleep and personal hygiene. Steps per day were logged on record sheets by each student. Data gathering spanned an entire week, and the results were sorted by alternate starting days, by activity for an entire week, by activity for only the weekdays of the one-week monitoring interval and for the two-day weekend. The statistical analysis included ANOVA, intra-class correlation (ICC) analysis, and regression analysis. The ICC analysis suggested that monitoring starting on Monday (ICC = 0.71; 95%CI (0.61–0.79)), Tuesday (ICC = 0.67; 95%CI (0.59–0.75)) or Thursday (ICC = 0.68; 95%CI (0.55–0.79)) improved reliability. The results of regression analysis also indicated that any starting day except Sunday is satisfactory as long as a minimum of four days of monitoring are used.

## 1. Introduction

The high rate of inactivity among adolescents and adults and the importance of regular physical activity (PA) to health outcomes have been well documented [1,2,3,4]. One key to improving the relationship between these important social issues is to increase the measurement-accuracy of PA metrics in controlled studies. Precise measurement is necessary to determine appropriate activity levels, to assess the relationship between PA and health and to plan and evaluate tailored intervention programs [5].

Early PA studies in this area suffered from numerous assessment and procedural problems. Researchers often relied on self-reporting measurement techniques that, while offering administrative and cost advantages, produced highly unreliable data. Welk [6] estimated the error rate associated with self-reporting tools to be 35%–50%.

More accurate assessment techniques emerged in the form of self-monitoring devices when public health concerns shifted from peak physical fitness to “lifestyle physical activity” [7,8]. The goal for this new initiative became moderate-intensity activity that could be accumulated within the framework of one’s daily routine.

The simplest and least expensive tool for measuring and predicting daily activity levels across various age groups is the pedometer [5,9,10]. This device is a motion sensor that measures steps, distance, time spent in activities and, in some cases, energy expended. However, the pedometers (YAMAX SW 701) we used did not store data and did not provide information on frequency, intensity, duration or type of activity at a certain point in time. Crouter *et al.* [11] indicate that, in general, pedometers are most accurate for assessing steps, less accurate for assessing distance, and least accurate for assessing kilocalories.

Additional tools for the assessment of PA are accelerometers that record both volume and intensity of PA, most often in activity counts, and also register the time spent at moderate to high intensity PA [5]. For adults, 3–5 days of monitoring PA is recommended for an estimate of habitual physical activity [12]. However, for older adults, at least 6 days of data collection are required to obtain a reliable PA estimate [13]. In contemporary situations, there is dominant usage of the three-dimensional recording accelerometer, the Actigraph (ActiGraph, Pensacola, FL, USA), that measures both total and current energy expenditure [5,14,15]. Actigraph has demonstrated good reliability for measuring both counts and steps [16]. Moreover, wearing an Actigraph on the belt above the hip for PA monitoring is very simple and non-restrictive. Compared to the pedometer, however, the large disadvantage of Actigraphy is its higher cost [14].

Due to their low cost and good accuracy, pedometers are now widely used in PA-research to recognise, assess and predict the free-living exercise habits of different population subgroups. A major concern in these studies is identifying the validity and reliability of the tools used to collect activity data. Validity here refers to the accuracy of the research tool’s measurement and reliability refers to the consistency of the resulting measurements.

Literature on this subject is replete with studies using different data-gathering intervals to establish the reliability of pedometer-generated step data and to predict habitual activity patterns. However, there is not widespread agreement among researchers on two key issues: what is the optimal monitoring interval? and what is the most advantageous day of the week to begin monitoring? For adult subgroups, Gretebeck and Montoye [17] determined that a minimum of five to six days of monitoring is required to establish reliable estimates, and Rowe *et al.* [13] reported that six days of data were needed to achieve a reliable ICC value of 0.70 for an adult population. Conversely, Tudor-Locke *et al.* [18] reported that only three days of monitoring of adult subgroups are needed to achieve an ICC of 0.80, and Craig *et al.* [19] determined that two days of monitoring is sufficient for a five- to nineteen-year-old age group to project steps per day and that only a single day is needed to achieve an ICC of 0.70. Obtaining differences in physical activity within single days of the week, or work days and weekends [20,21,22,23] provided evidence that the variability of day to day physical activity is not constant [24]. Within the context of the reaction to unsealed pedometers leading to increased step counts [25], the number of days necessary for reliable monitoring may vary according to the day of the week the monitoring began. On the other hand, any estimate based on the compound symmetry assumption could underestimate the number of days needed to obtain a given level of reliability [24].

This lack of agreement is not surprising. Raines-Eudy [26] has noted that the reliability of monitoring devices and the data they generate differ across ethnic, socioeconomic, and cultural subgroups. From this view, the choice of university students as a representative subgroup of a healthy adult population seems to be a suitable alternative. Czech students comprise a subgroup of a healthy adult population whose PA level is not influenced by other health factors. The lack of agreement makes clear the need for more research aimed specifically at identifying the most efficacious monitoring interval and starting day of the week. This study adds clarity to the existing data by examining the influence of the study interval and starting day on data reliability.

The aim of this work is to evaluate the reliability of a weeklong PA monitoring of university students in terms of a distinct day of the week to begin monitoring and the number of days for monitoring.

- Describe the differences in the number of steps in light of the day monitoring begins
- Determine the most appropriate day to begin monitoring in accordance with the ICC
- Establish the number of days of monitoring necessary to predict the week-long PA regarding the day monitoring begins

## 2. Methods

### 2.1. Ethics

The current study was undertaken in the Czech Republic following approval by the Institutional Research Ethics Committee at Palacky University. Participation was voluntary, and no rewards or incentives were offered for participation. The participants were informed of the aims, objectives and methods of the study before physical activity (PA) monitoring started. Confidentiality and data anonymity were carefully maintained throughout the study.

### 2.2. Participants

This study was part of a statewide research project on the PA of adult residents in the Czech Republic. Universities from the regional capital cities of Olomouc, Ostrava, Brno, Liberec, Hradec Kralove, Plzen and Ceske Budejovice were chosen as study sites. During the period from 2008–2010, 906 students were randomly selected as potential participants. From these potential probands, 720 students agreed to participate (a response rate of 79.5%). From this group, 79 students were excluded from the sample for providing extreme step values (the number of steps reported per day ranged from a minimum of 1,000 to a maximum of 30,000 steps per day [27]). Participants were also excluded for providing incomplete data (weight, height, age, gender or step values on a monitoring day). The remaining 641 subjects comprised the final eligible sample. Although step count sets containing missing values for two days were not significantly different from a complete dataset [28], only complete data sets from respondents (step counts for all seven days) were used for the analysis.

### 2.3. Assessment of Physical Activity

Study participants wore a Yamax SW-701 pedometer (Yamax Corporation, Tokyo, Japan) continuously for 7 days and for least 10 h per day; only periods of sleeping and hygiene were excluded. It was recommended that the pedometers be worn on the right hip [11,29]. Participants could choose the first day of monitoring and were responsible for recording daily pedometer readings on log sheets.

### 2.4. Statistical Analysis

This study sought to answer two important questions, “how many days of monitoring are needed to reliably project habitual physical activity for a whole week?” and “which day of the week is most efficient to begin the monitoring interval?” Sample data were analysed using SPSS 19 software and several suitable statistical techniques. Determination of the most effective starting day was made using ANOVA and the LSD post hoc test as well as Inter-Class Correlation (ICC (3,7); two-way mixed model, average measure). ICC was used to measure the predictive reliability of the pedometer-generated data across different starting days. Procedurally, this was accomplished by computing ICC coefficients for an entire week of data and then sorting the results by different starting days.

Determination of the most efficient monitoring interval for predicting weeklong physical activity was made using Stepwise Regression. The focus here was on the adjusted R^2^ (attempts to produce a more realistic value than the estimated R). A significance level of 0.05 was used in these analyses. The contribution of the extension of the monitoring interval was assessed based on the R square change according to hierarchical regression. The data set was tested for multicollinearity using the collinearity diagnostics in SPSS. The procedure used was straightforward. Different interval periods were regressed to physical activity data to identify which interval produced the highest predictive value.

## 3. Results

The data provided by the random sample of 641 college students from seven regional universities in the Czech Republic represented a broad range of young adults. Males constituted 49.7% (n = 319) of the sample and females 50.3% (n = 322); their ages ranged from 18 to 28 years (mean 21.33 years, SD = 1.65). BMI values varied widely from 17 to 35 kg/m^2^ (mean 22.36 kg/m^2^, SD = 2.35) with 89.5% of the sample exhibiting a normal BMI, 10% were considered overweight (BMI ≥ 25) and 0.5% obese (BMI ≥ 30) [30].

The mean PA level (defined as number of steps) for different starting days and over a one-week monitoring interval are shown in Table 1. The results are separated into activity for the entire week, for only the weekdays of the one-week monitoring interval, and for the two-day weekend. The ANOVA test indicated that the only statistically significant difference in mean steps per week, using a specific starting day, was between Sunday and Tuesday (F = 2.52; *p* = 0.02). The LSD post-hoc test confirmed that initiating monitoring on Sunday produced higher activity levels for the week than initiating on Tuesday.

**Table 1:** Mean number of steps/day averaged across an entire week stratified by the “starting day”.

Initial monitoring day		Entire week		Weekdays		Weekend	
	n	M	SD	M	SD	M	SD
Monday	99	12,110	3,191	12,829	3,519	10,329	4,121
Tuesday	151	11,409	3,225	11,991	3,669	9,956	3,904
Wednesday	155	11,876	3,086	12,671	3,414	9,889	4,360
Thursday	65	11,842	2,953	12,685	3,128	9,733	3,892
Friday	97	11,880	2,919	12,318	3,126	10,782	4,236
Saturday	17	10,917	2,761	10,677	3,098	11,517	3,618
Sunday	57	13,119	2,825	13,870	3,096	11,241	4,107

Note: M—mean; SD—standard deviation.

However, there were no statistically significant differences in activity levels when monitoring began on any of the other five days. The large differences in the number of subjects between these two days and the high standard deviation on both days reduced the evidentiary value of this comparison. The ANOVA test also indicated the absence of a statistically significant difference in the weeklong activity levels of the male and female students.

In this study, the ICC statistic describes the relationship between the initial monitoring day and the group activity data. As shown on Table 2, the highest ICC coefficients (reliability measures) were associated with monitoring intervals starting on Monday (0.71), Tuesday (0.67) and Thursday (0.68) with only the Monday coefficient meeting the minimum standard of >0.70 suggested by Trost *et al.* [31]. Given the relatively weak values for starting days other than Monday, the data show no conclusive evidence on which day of the week is the most efficient day to begin monitoring PA.

**Table 2:** Intra-Class Correlations [ICC (3,7)] and 95% confidence interval by starting day.

Initial monitoring day	n	ICC	95% Confidence Interval	
			Lower Bound	Upper Bound
Monday	99	0.71	0.61	0.79
Tuesday	151	0.67	0.59	0.75
Wednesday	155	0.61	0.51	0.69
Thursday	65	0.68	0.55	0.79
Friday	97	0.53	0.37	0.66
Saturday	17	0.55	0.12	0.81
Sunday	57	0.51	0.28	0.68

An anomaly in the results is shown in Table 1 and Table 2. The data in Table 1 indicate the highest level of PA over the entire week was achieved when the monitoring interval began on Sunday. The lowest ICC coefficient, however, was associated with Sunday as the starting day.

The results from the regression analysis are shown in Table 3. As expected, the predictive value, measured by R-squared, adjusted R-squared and the standard error of the estimate, increased with the monitoring interval. When the monitoring interval was increased to four days, the adjusted R-squared value increased to 0.756, and the standard error of the estimate declined to 1,526.8.

**Table 3:** Regression statistics for selected monitoring intervals.

Monitoring interval	R	R^2^	Adjusted R^2^	Standard Error of the Estimate
One day	0.53	0.28	0.28	2,622.15
Two days	0.70	0.49	0.49	2,209.30
Three days	0.81	0.65	0.65	1,828.58
Four days	0.87	0.76	0.76	1,526.82
Five days	0.92	0.85	0.84	1,218.57
Six days	0.97	0.94	0.94	788.48
Seven days	1.00	1.00	1.00	0.00

The results from the regression analysis for selected starting days and monitoring intervals are shown in Table 4. Except for the monitoring when Sunday was the starting day, the adjusted R-squared value for the four day monitoring interval was >0.70. The three day monitoring interval had an R-squared value >0.70 only when the starting days were Tuesday, Thursday, Saturday or Sunday.

**Table 4:** Adjusted R-squared values by starting day and monitoring interval

Monitoring interval	Starting day						
	Mon	Tue	Wed	Thu	Fri	Sat	Sun
One day	0.36	0.35	0.27	0.49	0.25	0.38	0.13
Two days	0.54	0.62	0.47	0.58	0.39	0.42	0.40
Three days	0.65	0.72	0.67	0.77	0.56	0.73	0.79
Four days	0.78	0.80	0.79	0.84	0.71	0.84	0.61
Five days	0.89	0.89	0.84	0.89	0.82	0.89	0.75
Six days	0.95	0.95	0.94	0.95	0.94	0.93	0.91
Seven days	1.00	1.00	1.00	1.00	1.00	1.00	1.00

* All cells depict Adjusted R-squared values from the regression analysis.

Table 5 shows how the addition of each interval added to the variance accounted for by the previous interval. From the results, it is evident that for one day of monitoring, the R square change was 0.28, which was statistically significant (*p* < 0.001). For two days of monitoring, there was an increase in the R square value of 0.21 (*p* <0.001). A third day of monitoring significantly added approximately 16% of the variance, and a fourth day of monitoring added 11% of the variance. A fifth, sixth or seventh day of monitoring added relatively little (less than 10%) of the variance.

**Table 5:** R-squared changed values by starting day and by adding days to the monitoring interval.

Predictors	R-square change	*p*
Starting day	0.01	0.067
Starting day, 1st day	0.28	<0.001
Starting day, 1st day, 2nd day	0.21	<0.001
Starting day, 1st day, 2nd day, 3rd day	0.16	<0.001
Starting day, 1st day, 2nd day, 3rd day , 4th day	0.11	<0.001
Starting day, 1st day, 2nd day, 3rd day , 4th day, 5th day	0.09	<0.001
Starting day, 1st day, 2nd day, 3rd day, 4th day, 5th day, 6th day	0.08	<0.001
Starting day, 1st day, 2nd day, 3rd day, 4th day, 5th day, 6th day, 7th day	0.064	<0.001

Table presented R-squared change from the hierarchical regression analysis; dependent variable is weekly step counts; *p*—significance F change from hierarchical regression.

## 4. Discussion

The pedometer is widely used by researchers and practitioners to measure step-counts, to establish activity indices and to prescribe behavioural guidelines. Despite its increased use, there remains the need for greater precision in the measurements used to evaluate pedometer-generated data. Improved accuracy would lead to both more reliable predictions of habitual activity and more effective intervention programs. 

An important factor affecting the reliability of extrapolating activity patterns from empirically derived data is the degree of variability in the data [24]. Logically, as variability increases, the monitoring interval used to gather evidence to support predictions should also increase. This simple logic, however, begs the question of what length of monitoring interval is needed to insure reliability. Shortening the monitoring interval reduces research costs, but in so doing, reliability is sacrificed. The goal, then, is to optimise the trade-off between the monitoring interval length and an acceptable level of reliability.

The evidence from available research on monitoring intervals and starting day is inconclusive. The often-identified reason for this inconclusiveness is inherent differences in the characteristics of the population. For example, Raines-Eudy [26] suggests that research findings will differ across ethnic, socioeconomic and cultural subgroups. Stone *et al.* [32] go further and suggest the validity and reliability of monitoring devices depends on both the population and the setting in which they are used. 

### 4.1. The Pedometer

The pedometer is the ideal device for monitoring and measuring free-living, ambulatory physical activity. It is widely used in cross study comparisons of different populations and in a variety of clinical and community intervention programs. This scientific tool offers the advantages of low cost, ease-of-application and data production that are easily comprehended, objective and quantifiable [33]. Despite these advantages, the pedometer has limitations. 

The most obvious limitation, but one unrelated to research designs similar to this study, is its limited use. The pedometer is simply not practical for measuring a variety of aerobic and anaerobic physical activities where step count is not the target. Conversely, other limitations are specific to step-count studies. Accuracy varies between manufacturers’ models making the absence of uniformity a consideration for inter-study comparison. Additionally, step-count data generated outside a controlled laboratory environment, such as in free-living studies, are susceptible to upside bias. Because the readout is readily visible to participants, it can easily influence artificially high levels of activity. Likewise, when participants are responsible for personally logging activity, the results may be exaggerated and susceptible to upside bias.

### 4.2. Daily Step Count

There are a number of prominent studies on pedometer-measured PA that produced different results. For instance, Tudor-Locke *et al.* [18] found that Sunday produced the lowest average step counts for the week, while the data from this study show the largest average numbers of steps were taken on Sunday. Additionally, this study revealed a statistically significant difference between Sunday and Tuesday activity levels. Step counts could also be influenced by reactivity, due to use of unsealed pedometers. This could cause increased step counts, especially for a one week long period [25]. The negligible difference between these two studies (between results from our study and Tudor-Locke *et al.* [18]) appears to be attributable to the age difference between the two population subgroups. The sample used in this study was drawn from a group of university students, many of which could be members of sports clubs. Sunday is the day on which sports clubs often hold competitive events, and the activity levels of this subgroup of students could have a positive influence on the data.

Male and female step counts also differed between this study and others. Somewhat surprisingly, the data in the present study showed no significant difference between male and female activity levels over an entire week, for weekdays or for weekend days. Other studies, such as Frömel *et al.* [34], Behrens and Dinger [35], and Vasickova *et al.* [36], found statistically significant differences between male and female step counts over different monitoring intervals. These differences may be explained by the composition of the sample drawn for this study. Highly active students made up a sizable portion of the sample, while obese students comprised only 10% of this subgroup. This difference supports the contention of Raines-Eudy [26] that it is important to identify subgroup characteristics when analysing results.

### 4.3. Initial Measurement Day

Intra-Class Correlation (ICC) is a commonly used statistical technique for identifying the reliability of physical activity patterns in pedometer-generated data [37]. Blind reliance on suggested ICC coefficient cut-off scores is not, however, advisable. For example, Trost *et al.* [31] and Bot *et al.* [38] recommend an ICC cut-off value of 0.70 for predictive reliability while Tudor-Locke *et al.* [18] and Traub [39] suggest 0.80. These values are subjective judgments that lack the support of rigorous testing of extensive empirical data. It is what Atkinson and Nevill [37] refer to as a “research gap”. Moreover, as discussed by Atkinson and Nevill [37], there are different methods of applying intra-class correlation and each method may produce different results for the same data.

Baranowski *et al.* [40], Levin *et al.* [41], and Matthews *et al.* [42] suggest an even more cautionary limitation, which is that the assumption of compound symmetry in the ordinary least squares method of the ICC model may not be appropriate for daily pedometer-generated data. Compound symmetry refers to the assumption that the variance and covariance in the data are the same across the days and, therefore, the correlations between days are comparable. Thus, when the data do not fit this assumption, it is not appropriate to summarise the reliability estimate with a single value.

In the present study, the highest level of step counts over the entire week was achieved when the monitoring interval began on Sunday. The lowest ICC coefficient, however, was associated with the Sunday starting day. Again, this anomaly has two possible sets of explanations. First, the potential effects of the small number of cases and relatively large standard deviation are associated with the Sunday data. The second explanation is the composition of the sample population in which many of the students could be members of sports clubs, which often compete on Sundays.

### 4.4. Monitoring Interval

The data from this study are consistent with parallel studies and clearly support the thesis that activity patterns can be reliably predicted for monitoring intervals of less than one week. Exactly how much less has not been definitively established. For example, Vincent and Pangrazi [43] studied a sample of children ranging in age from seven to twelve years and found ICC values ranging from 0.65 for two days of monitoring to 0.87 over 8 days. The data in the present study suggest at least four but no more than five days of monitoring are needed to reliably predict an activity pattern for a population of college age young adults. The adjusted R-squared value (0.756) indicates that 76% of the variability in physical activity is explained by the 4-day interval. There is not, however, total agreement on what constitutes an acceptable minimum. Baranowski *et al.* [40] for example, suggested that an adjusted R-squared value of at least 0.80 is necessary for predictive reliability. Using this cut-off value, a monitoring interval of five days is suggested. Depending on which minimum standard for weeklong predictive reliability is used, the conclusion here is obvious. If the Trost recommendation of >0.70 is accepted [31], then a four-day monitoring interval starting on any day other than Sunday can be used to extrapolate week-long PA. Alternatively, if Baranowski and Moor’s recommendation is used as the cut-off value, then a five-day monitoring interval starting on any day other than Sunday would be used [44]. The present study also showed that the highest contribution of variance was accounted for by the first four days of monitoring. Nevertheless, care is necessary when presenting the results because the estimate is based on the compound symmetry assumption, and it could underestimate the number of days needed to obtain a given reliability [24]. Tudor-Locke *et al.* [18] found that only a three-day monitoring interval was needed when dealing with an adult population, while Gretebeck and Montoye [17] recommend five to six days for adult populations. What these findings seem to suggest about the length of the monitoring interval is that age and lifestyle characteristics of the population under study are possibly more important than using rigidly defined statistical standards.

### 4.5. Limitations

Despite the large amount of analysed data (641 students x 7 days), this study has definite limits. All of the students from universities are included in the analysis without regard to their area of study. Thus, students from faculties with a physical education focus were included, and their data could potentially have distorted the final step counts. The pedometers lacked a blinded display, which could lead to greater physical activity as it could serve as a motivational device. The reactivity to the unsealed pedometers could cause increased step counts, and this effect could last for a period of one week [25]. Step counts from the pedometers do not give a measure of the intensity of the PA. Furthermore, participants also registered the number of daily steps into the record charts; this could make the pedometers serve as a “semi-objective” tool. There were a very small number of participants, who finished monitoring their step counts on Saturday; this could also have influenced the results. Another characteristic, which could have influenced the results, was the small representation of overweight or obese individuals (10%). Finally, the environment and the facilities at the different colleges and universities could have influenced the movement of the students; this potential influence was not analysed in this work.

## 5. Conclusions

These data add to the body of pedometer-driven, self-managed research. They suggest that, while there are significant differences between the starting days examined in different studies, there is little practical difference. In general, the specific characteristics of the population appear to be the most important determinant of which days are not effective starting points for monitoring; there is little practical difference between the remaining days of the week.

The data from this and parallel studies are also in general agreement that monitoring intervals of less than one week in length have predictive reliability. Again, the defining characteristics of the sample population, especially age and lifestyle, appear to be the most important determinants of the appropriate length of the monitoring interval. Lastly, the apparent link between population characteristics and research-design variables suggests that this is a fertile area for future research.
